# Recombinant TLR5 Agonist CBLB502 Promotes NK Cell-Mediated Anti-CMV Immunity in Mice

**DOI:** 10.1371/journal.pone.0096165

**Published:** 2014-05-30

**Authors:** Mohammad S. Hossain, Sampath Ramachandiran, Andrew T. Gewirtz, Edmund K. Waller

**Affiliations:** 1 Department of Hematology and Medical Oncology, Division of Stem Cell and Bone Marrow Transplantation, Winship Cancer Institute, Emory University School of Medicine, Atlanta, Georgia, United States of America; 2 Department of Biology, Georgia State University, Atlanta, Georgia, United States of America; Charité, Campus Benjamin Franklin, Germany

## Abstract

Prior work using allogeneic bone marrow transplantation (allo-BMT) models showed that peritransplant administration of flagellin, a toll-like receptor 5 (TLR5) agonist protected murine allo-BMT recipients from CMV infection while limiting graft-vs-host disease (GvHD). However, the mechanism by which flagellin-TLR5 interaction promotes anti-CMV immunity was not defined. Here, we investigated the anti-CMV immunity of NK cells in C57BL/6 (B6) mice treated with a highly purified cGMP grade recombinant flagellin variant CBLB502 (rflagellin) followed by murine CMV (mCMV) infection. A single dose of rflagellin administered to mice between 48 to 72 hours prior to MCMV infection resulted in optimal protection from mCMV lethality. Anti-mCMV immunity in rflagellin-treated mice correlated with a significantly reduced liver viral load and increased numbers of Ly49H+ and Ly49D+ activated cytotoxic NK cells. Additionally, the increased anti-mCMV immunity of NK cells was directly correlated with increased numbers of IFN-γ, granzyme B- and CD107a producing NK cells following mCMV infection. rFlagellin-induced anti-mCMV immunity was TLR5-dependent as rflagellin-treated TLR5 KO mice had ∼10-fold increased liver viral load compared with rflagellin-treated WT B6 mice. However, the increased anti-mCMV immunity of NK cells in rflagellin-treated mice is regulated indirectly as mouse NK cells do not express TLR5. Collectively, these data suggest that rflagellin treatment indirectly leads to activation of NK cells, which may be an important adjunct benefit of administering rflagellin in allo-BMT recipients.

## Introduction

CMV infection is usually asymptomatic in immune-competent healthy individuals, but may cause severe disease in immune-compromised BMT, HIV-infected AIDS, and elderly patients [1]. Interstitial pneumonitis is the most serious manifestation of CMV disease causing 30–48% patient mortality [2]. While numerous anti-viral drugs are available, the occurrence of drug-resistant CMV strains increases treatment-related complications in these patients [3]. Naturally, CMV infection is controlled by both innate and adaptive immunity [4], [5]. Tabeta et al showed that innate anti-mCMV immunity is mostly controlled TLR9- and TLR3-dependent signaling during the early phase of infection, and others have shown that flagellin enhances the activation and proliferation of NK cells [6], [7]. We have previously shown that prophylactic administration of native flagellin, a TLR5 agonist protein extracted from the flagella of *Salmonella typhimurium*, protected allo-BMT recipients both from GvHD and lethal CMV infection [8]. Flagellin has diverse immune-modulatory activity on both innate and adaptive immunity in mice and humans [4], [9], [10] [5], [11], [12]. The highly purified cGMP grade rflagellin variant CBLB502 is exceptionally stable, less toxic and less immunogenic than native flagellin [13], [14]. Administration of rflagellin reduced radiation-induced toxicity in mice and non-human primates [13], [14], but the role of flagellin-TLR5 interactions in the anti-MCMV immunity of NK cells has not been described. The present study was undertaken to elucidate the mechanism by which rflagellin-TLR5 regulates NK cells immunity in mice infected with a lethal inoculum of mCMV.

NK cells are a major component of innate immunity, and are critical to the early immune response to mCMV infection [15]–[17]. The mCMV infection leads to activation of NK cells, and activated NK cells directly kill CMV-infected target cells, reducing viral replication [18]. In B6 mice, NK cells control mCMV infection through a number of activating receptors, including NKG2D, NKp46, NK1.1 (NKR-P1C), Ly49D and Ly49H. Down-stream activation via these receptors is initiated by the cytoplasmic immunoreceptor tyrosine-based activation motif (ITAM) associating with the DAP12 adaptor protein complex [19]–[23]. 2B4 (CD244, a non-MHC binding receptor), another surface marker of NK cells, also induces both activation and inhibitory responses depending on the phosphorylation of the cytoplasmic tyrosine motifs. The activated isoform of 2B4 induces NK cell activation via coupling with the NKG2D-DAP10 complex [19]. The killer cell lectin-like receptor G1 (KLRG1) is known to be an inhibitory surface marker for NK cells, but KLRG1 expression is also required for maturation, activation and homeostatic proliferation of NK cells [24].

In this study, we investigated how rflagellin binding to TLR5 contributes to NK cell activation and the anti-mCMV immunity of NK cells in B6 mice. We observed that a single dose of rflagellin administered 48 hours prior to mCMV infection protected mice from a lethal dose of mCMV. rFlagellin treatment led to significantly reduced viral load in the liver along with significantly increased numbers of mature, activated cytotoxic Ly49H- and Ly49D-expressing NK cells compared with the PBS-treated control mice. We found that tonic flagellin signaling through TLR5 is necessary for optimal activation of NK cells immune response to mCMV infection. Together, these data provide new mechanistic insights about the effects of rflagellin-TLR5 binding on NK cell activation against mCMV infection.

## Materials and Methods

### Mice

C57BL/6 (B6) mice were purchased from Jackson Laboratories (Bar Harbor, ME). TLR5^−/−^ knock out (KO) mice with B6 background were bred at the Emory University animal facility. All experimental procedures conformed to *the Guide for the Care and Use of Laboratory Animals*, and were approved by the Emory University Institutional Animal Care and Use Committee (IACUC Protocol # 2001896).

### Production of rflagellin and administration to mice

rFlagellin is a pharmacologically improved recombinant derivative of *Salmonella* flagellin, in which the central variable segments (domains D2 and D3) have been deleted and the structural elements required for TLR5 signaling (domains D0 and D1) are retained. The highly purified cGMP grade rflagellin variant CBLB502 is produced by Cleveland Biolabs, NY as previously described [13], [25]. Briefly, the rflagellin cDNA (from *Salmonella dublin*) is overexpressed in *E. coli* and a fusion protein of flagellin with an N-terminal His_6_-tag is purified to homogeneity by a combination of Ni-NTA chromatography and FPLC-based gel-filtration. The final product (>95% pure by SDS-PAGE) is purified from residual LPS by passing though detoxigel (Pierce, Rockford, IL). This purification process allowed us to obtain >100 mg of pure rflagellin from 6L of bacterial culture. We obtained rflagellin from Cleveland Biolabs through a collaborative agreement between Emory University and Cleveland Biolabs. The aliquots of rflagellin were stored at −80°C and reconstituted in ice-cold 0.1% Tween-80 in PBS (PBS). A single dose of 25 µg/0.2 ml PBS was injected in mice i.p 48 hours before mCMV infection or otherwise stated in the experiments.

### MCMV infection

rFlagellin-treated B6 or TLR5 KO mice were infected with non-lethal (1×10^5^ PFU/mouse i.p) or lethal [1×LD50 (i.e., 0.5×10^6^ PFU/mouse i.p) or more] doses of salivary-gland-passed Smith strain mCMV (a gift from Dr. H. Yushida, Saga University, Japan).

### Liver viral load determination

Livers were aseptically harvested on days 3 and 10 post mCMV infection. The mCMV pfu per liver was determined as previously described [26]. Briefly, collected liver was homogenized and centrifuged, and serially diluted supernatants were added to confluent monolayers of 3T3 cells in 24-well tissue culture plates. After incubation for 90 minutes at 37°C, ∼1 mL 2.5% methylcellulose in DMEM (10% FBS) was added to each well of treated 3T3 monolayers and incubated for an additional 4 days at 37°C. mCMV pfus were directly counted under a light microscope (Nikon, Melville, NY) after removing the methylcellulose and staining the 3T3 cells with methylene blue.

### Isolation and measurement of leucocytes from the spleens of experimental mice

Mice were sacrificed, splenocytes were harvested, single cell suspensions were prepared and total nucleated cells per spleen were counted by using a fluorescent microscope as previously described [8].

### In vivo depletion of NK cells

NK cells were depleted by using rabbit antiserum against asialo GM1 (anti-asialo GM1, Wako Chemicals) in B6 mice as previously described [26] with a slight modification. 1 vial of anti-Asialo GM1 was reconstituted in 6 ml PBS. 0.2 ml of reconstituted anti-asialo GM1 was further diluted to 0.5 ml in PBS and injected intraperitoneally in B6 mice on 4, 3 and 1 day prior to mCMV infection (5×10^5^ pfu/mouse i.p). The three doses of anti-asialo GM1 selectively depleted blood CD3-NK1.1+ cells by >99% as determined by flowcytometry (Figure S2) before mCMV infection.

### Measurement of NK cells cytotoxic activity

NK cell cytotoxic activity was determined by using standard 4 hour ^51^Cr-release assay as previously described [20]. Briefly, splenocytes were harvested from rflagellin- and PBS-treated control mice on day 0, 1, 2, 3 and 8 after mCMV infection. NK-sensitive Yac-1 target cells were labeled with 37 MBq Na^51^CrO_4_ at 37°C for 90 min and washed three times with RPMI 1640 complete medium. The labeled target cells (1×10^4^) were co-cultured with whole splenocytes (effector cells) at various effector: target (100∶1, 50∶1, and 25∶1) ratios in a final volume of 0.2 ml fresh RPMI 1640 complete medium in 96-well U-bottomed tissue culture plates for 4 hours at 37°C. The labeled Yac-1 target cells (1×10^4^) co-cultured with either only complete media or 1% Triton X were used for minimum and maximum release, respectively. The amount of ^51^Cr released in the 0.05 ml supernatant/well was determined by a well-type gamma counter (beta liquid scintillation counter; EG&G Wallac, PerkinElmer, Ontario, Canada). Specific cytotoxicity was calculated as follows: % ^51^Cr release = 100×(cpm experimental - cpm spontaneous release)/(cpm maximum release - cpm spontaneous release).

### Measurement of TLR5 expression in FACS sorted NK cells and TLR5-transfected cell lines

To determine the TLR5 expression by NK cells, CD3-NK1.1+ NK cells were sorted by FACS from the spleens of immunologically-naïve B6 mice. Total RNAs were harvested from sorted NK cells (>4.0×10^6^ sorted NK cells, >95% purity), HEK-Blue-mouse TLR5 transfected 293 cells and HEK-Blue-Null2-k 293 Cells (InvivoGen, CA) using RNeasy kit (Qiagen). Single strand cDNA was synthesized using 1 µg of total RNA, and QuantiTect Reverse Transcription Kit (Qiagen) in a total volume of 20 µL. 5 µl of cDNA reaction was used in the PCR reaction with primers specific for TLR5 (forward primers 5 -GGA CAC TGA AGG ATT TGA AGA TG-3 and reverse primers 5 -GGA CCA TCT GTA TGC TTG GAA TA-3) [27] or GAPDH as a control. Samples were amplified by 35 cycles and loaded on to a 1.5% agarose gel and subjected to electrophoresis. Specific bands were detected by staining with ethidium bromide.

### Serum cytokines measured by Luminex assay

Serum was harvested on day 0, 2 and 3 after MCMV infection from rflagellin- and PBS-treated B6 mice. Mouse 26-plex and IFN-α/β kits were purchased from Affymetrix Inc (Santa Clara, CA) and the Luminex assay (Luminex Corp., Austin, Texas) was performed in a blinded fashion by the Immunology Core Laboratory at Stanford University (Stanford, CA) according to the manufacturer's recommendations. All samples were assayed in a single batch, and each sample was measured in duplicate. Plates were read using a Luminex 200 instrument (Luminex Corp) as previously described [28].

### Flow cytometry

The NK cells (CD3-NK1.1+) were determined by staining of splenocytes harvested from B6 mice with anti-mouse mAbs to CD3 and NK1.1. The activation status of NK cells was determined by staining the cells with mAbs to ICOS-1, CD69, KLRG1, 2B4, Ly49G2, Ly49C/H, Ly49D, Ly49H, CD122, CD11b, CD27, CD107a, etc. All antibodies were purchased from either BD Pharmingen (San Jose, CA) or eBioscience. The frequencies of granzyme B and IFN-γ producing NK cells were determined by staining for intracellular cytokines after 4 hours of *in vitro* stimulation of whole splenocytes with PMA-calcium ionomycin with Golgi Plug cocktail (BD Pharmingen) as described before [8]. The stained cells were acquired by FACScanto (Becton Dickinson, San Jose, CA) and analyzed by FlowJo software.

### Statistical analyses

Student's *t*-test and Log Rank test were used to determine the statistical significance of the acquired data. Differences between groups were considered statistically significant when *p* value<0.05 was obtained.

## Results

### Prophylactic rflagellin administration induced strong anti-MCMV immunity

**Figure 1:**
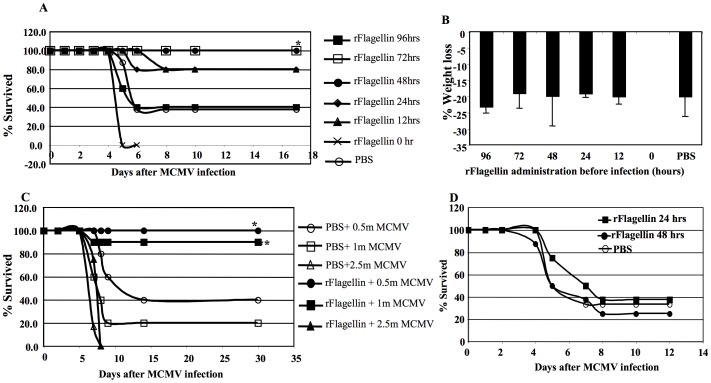
Prophylactic rflagellin administration protected mice from lethal mCMV infection. A. A total of 5 groups WT B6 mice were treated with 25 µg rflagellin/mouse i.p 96, 72, 48, 24 or 0 hours before a lethal dose (2×LD50, 1×10^6^ pfu/mouse i.p) of mCMV infection. Control mice were treated with PBS only 48 hours before the same lethal dose of mCMV infection. Infected mice were monitored every day to record for mortality. The % survival recorded until day 17 after mCMV infection is shown. The symbol “*” indicates the p value<0.05, Log Rank Test of groups rflagellin 48 hrs or 72 hrs vs PBS-treated group. B. The % weight loss measured on day 5 after mCMV infection. All mice receiving rflagellin at 0 hours after mCMV infection died before day 5 after mCMV infection and the % weight loss data of this group was not available. 5–10 mice were used per group. C. WT B6 mice were treated with a single dose of rflagellin 25 µg/mouse or 0.2 ml PBS i.p. 48 hours later mice were infected either with 0.5×10^6^, 1×10^6^ or 2.5×10^6^ mCMV pfu/mouse i.p. The % survival on day 28 after mCMV infection is presented. 10 mice were used per group. The % survival recorded until day 17 after mCMV infection is shown. The symbol “*” indicates the p value<0.05, Log Rank Test of while compared with the survival data of rflagellin-treated mice vs corresponding mCMV infection dose in PBS-treated mice. 5–10 mice were used per group. D. WT B6 mice were infected with 0.5×10^6^ mCMV pfu/mouse i.p. A single dose of rflagellin (25 µg/mouse) was injected i.p 24 or 48 hours after mCMV infection. Control mice were injected with 0.2 ml PBS i.p 24 hours after mCMV infection. Survival of mice after mCMV infection was monitored each day and % survived mice until 12 days after infection is presented. 6–8 mice were used per group.

We have previously shown that prophylactic administration of two doses of native flagellin (50 µg/mouse i.p) 3 hours before irradiation and 24 hours after transplant protected allo-BMT recipients from GvHD by inducing transient immunosuppression of donor T cells. Paradoxically, flagellin treatment also protected allo-BMT recipients from lethal mCMV infection [8]. Like native flagellin, rflagellin also protected allo-BMT recipients from GvHD in a similar fashion, with the optimal i.p dose between 25 µg to 50 µg/mouse (our unpublished data). To study the mechanism by which flagellin confers protection from mCMV infection without the immunological complexity created by allo-transplantation, we studied the effects of rflagellin-treatment in non-transplanted WT B6 mice infected with lethal dose of mCMV. First, to confirm whether prophylactic rflagellin administration could enhance innate and adaptive immune responses to mCMV, WT B6 mice were treated with 25 µg rflagellin i.p or PBS 96, 72, 48, 24, 12 or 0 hours prior to infection with a lethal i.p dose (1×10^6^ pfu, 2×LD50) of mCMV. All mice that received rflagellin 72 or 48 hours prior to MCMV infection survived (p<0.05 compared with PBS-treated control mice) to 17 days post-infection, a time at which CMV-induced pathogenicity had resolved. In contrast, mice receiving rflagellin at earlier (96 hours) or later times (24 or 12 hours before mCMV infection) had 40%, 80% and 80% survival, respectively (Figure 1A). Interestingly, all mice receiving rflagellin at the same time as mCMV infection (0 hour) died within 5 days (Figure 1A). Control mice treated with PBS 48 hours before mCMV infection had 37.5% survival (Figure 1A).

We used the weight-loss of individual mice 5 days after infection as a measure of mCMV pathogenicity. Weight losses were similar among all rflagellin- and PBS-treated control groups, except the mice that received rflagellin simultaneously with mCMV infection (0 hour) had all died before day 5 post mCMV infection and the data were not available (Figure 1B). Next, we determined the relative effectiveness of prophylactic rflagellin administration by infecting rflagellin-treated mice 48 hours later with a range of mCMV doses. Both rflagellin- and PBS-treated control mice receiving a very high dose, 2.5×10^6^ pfu/mouse (i.e., 5×LD50) died within 7 days after mCMV infection (Figure 1C). The rflagellin-treated mice receiving 0.5×10^6^ pfu/mouse (i.e., 1×LD50) or 1×10^6^ pfu/mouse (i.e., 2×LD50) had 100% and 90% survival (p<0.05 compared with the corresponding PBS-treated groups), respectively (Figure 1C). In contrast, only 40% and 20% of PBS-treated mice survived after receiving 0.5×10^6^ pfu/mouse or 1×10^6^ pfu/mouse of mCMV, respectively (Figure 1C). Second, we determined the effect of 25 µg rflagellin/mouse i.p administered 24 or 48 hours *after* a lethal dose of mCMV (0.5×10^6^ pfu/mouse i.p) in WT B6 mice. Similar to PBS-treated control mice, WT B6 mice receiving rflagellin 24 or 48 hours after mCMV infection had less than 40% survival (Figure 1D). These data suggest that a single intraperitoneal dose of rflagellin administered 48 hours before mCMV infection yields the maximal effect on anti-mCMV immunity. Additionally, administration of rflagellin 25 µg/mouse i.p did not cause any noticeable toxicity as determined by weight loss within 48 hours (Figure S1A) and prevented weight loss typically seen following low dose mCMV infection (1×10^5^ pfu/mouse i.p) (Figure S1B). In contrast, PBS-treated control mice had significant weight lost by day 3 after mCMV infection compared with the weight prior to infection (Figure S1C).

**Figure 2:**
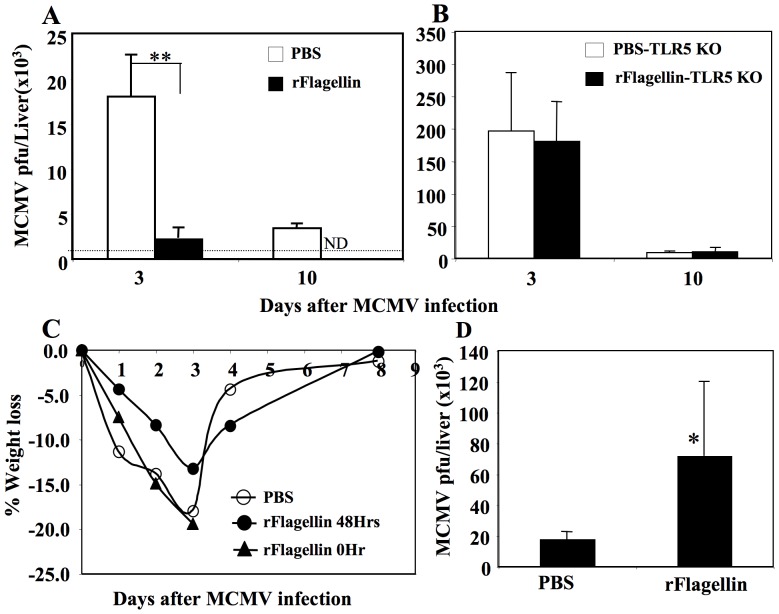
Prophylactic rflagellin administration reduced liver mCMV load in WT B6 mice. WT B6 and TLR5 KO B6 mice were given 25 µg rflagellin or 0.2 ml PBS i.p 48 hours before a sub-lethal dose (1×10^5^ pfu/mouse i.p) of mCMV. Mice were sacrificed on day 3 and 10 after mCMV infection and viral load per liver was determined as described in Materials and Methods. A. Virus titer in livers of rflagellin- and PBS-treated WT B6 mice. B. Virus titer in livers of rflagellin- and PBS-treated TLR5 KO B6 mice. The data are the representative of three similar experiments using 5 mice per group at each time point. C. WT B6 mice were given 25 µg rflagellin/mouse (rFlagellin 48 Hrs, closed circle) or 0.2 ml PBS (open circle) i.p 48 hours before or at the same time as (rFlagellin 0 Hr, closed triangle) a sub-lethal mCMV infection (1×10^5^ pfu/mouse i.p). Weights of individual mice were measured on day 0, 1, 2, 3, 4 and 8 days after mCMV infection. Percent weight changes per group of experimental mice are presented. D. Mice receiving rflagellin (rFlagellin 0 Hr) during mCMV infection became dehydrated and hunched and were sacrificed on day 3 after mCMV infection and liver viral load was determined. The data are the representative of two similar experiments using 5 mice per group. The symbols “*” and “**”represent the *p* values<0.05 and <0.005, respectively, Students *t*-Test.

Since the liver is one of the primary target organs for mCMV infection in mice [29] and anti-mCMV immunity is inversely correlated with viral load, we next determined the viral load in the liver of rflagellin- and PBS-treated mice on days 3 and 10 following a non-lethal (i.e., 1×10^5^ pfu/mouse i.p) dose of mCMV infection. Mice treated with rflagellin 48 hours before mCMV infection had significantly reduced viral load (**p<0.005) in the liver on day 3 and had faster liver viral clearance (not detectable, ND) on day 10 after mCMV infection compared with the PBS-treated control mice (Figure 2A).

Since flagellin is the only known ligand for TLR5 and rflagellin avidly binds TLR5 [13], we next confirmed the requirement for rflagellin-TLR5 immune interaction in anti-mCMV immunity by using TLR5 KO mice. TLR5 KO B6 mice had increased susceptibility to mCMV infection compared with the WT B6 mice (Figure S2A and S2B) with a LD50 of mCMV ∼2-fold less than in WT mice (Figure S2C). TLR5 KO mice were treated with rflagellin or PBS 48 hours before mCMV infection (i.e., 1×10^5^ pfu/mouse i.p) and viral load was determined on day 3 and 10 after mCMV infection. Both rflagellin- and PBS-treated TLR5 KO mice had similar liver virus titers on day 3 after mCMV infection (Figure 2B), but viral titers were ∼10-fold higher in TLR5 KO mice compared with PBS-treated WT mice (Figure 2A). These data suggest that endogenous signaling through TLR5 is important in protecting WT mice from mCMV infection. Additionally, mice treated with rflagellin prior to mCMV infection had less mCMV-induced pathogenicity (weight loss) compared with PBS-treated WT mice (Figure 2C). WT mice that received rflagellin at the same time as a sub lethal mCMV infection (i.e., 1×10^5^ pfu/mouse i.p) had more weight loss, appeared sick, and had to be sacrificed by day 3 post mCMV infection. Mice treated simultaneously with rflagellin and mCMV had higher liver viral loads on day 3 after mCMV infection compared with PBS-treated control mice (Figure 2D). Taken together, these data suggest that rflagellin initiates immune responses that require 1–2 days to become fully active in protecting mice from mCMV infection.

### Anti-mCMV immunity in rflagellin-treated mice mostly mediated by NK cells

**Figure 3:**
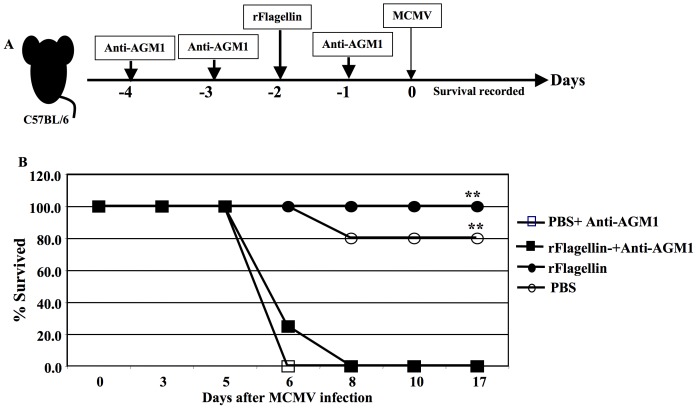
NK cells are required to induce enhanced early anti-mCMV immunity in rflagellin-treated mice. A). Experimental design of anti-asialo GM1 administration in WT B6 mice to deplete NK cells *in vivo*. Reconstituted anti-asialo GM1 in PBS and 0.5 ml was injected i.p to B6 mice on −4, −3 and −1 days of mCMV infection as described in Materials and Methods. Control WT B6 mice were injected with 0.5 ml PBS. 25 µg rflagellin was injected per mouse i.p 48 hours before mCMV infection in anti-asialo GM1-treated and or PBS treated WT B6 mice. All groups of treated mice were infected with a lethal dose (5×10^5^ pfu/mouse) of mCMV i.p on day 0. B). Survival data were recorded by observing mice every day or mice were euthanized having weight loss >25% following mCMV infection and percent survived mice of each group are presented. This experiments was performed once using 8 to 10 mice per group. The symbol “**”represents the *p* value<0.005, Log Rank Test (Kaplan-Meier estimator).

NK cells are the major component of innate immunity, and they play a key role in controlling mCMV infection [26]. To confirm the anti-mCMV immunity in rflagellin-treated mice is mediated by NK cell, we next depleted NK cells *in vivo* by administering anti-asialo GM1 before and after rflagellin treatment and infecting mice with a lethal dose (5×10^5^ pfu i.p) mCMV 48 hours after rflagellin treatment (Figure 3A). Anti-asialo-GM1 treated mice had >99% NK depletion (Figure S3). Interestingly, all mice treated with anti-asialo GM1 alone or with rflagellin died within 8 days following mCMV infection while rflagellin- and PBS-treated control mice had 100% and 80% survival, respectively (p<0.001 comparing PBS-treated group to treated groups) (Figure 3B). These data indicate that anti-mCMV immunity in rflgellin-treated mice is dependent upon the presence of NK cells.

### Prophylactic rflagellin administration enhanced anti-mCMV immunity by increasing the numbers of activated cytotoxic NK cells

**Figure 4:**
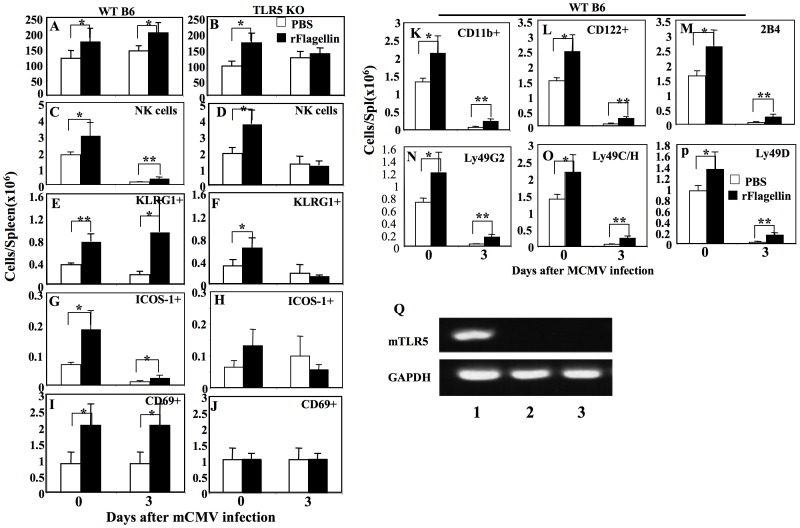
rFlagellin treatment increased NK cell activation in the absence and presence of mCMV infection. Splenocytes were harvested from rflagellin- and PBS-treated WT B6 and TLR5 KO B6 mice on day 0 and 3 after mCMV infection (1×10^5^ pfu/mouse i.p). A and B. Nucleated cells per spleen were determined from WT B6 and TLR5 KO B6 mice. C–J. Numbers of: CD3-NK1.1+ NK cells (C and D); KLRG2+ NK cells (E and F); ICOS-1+ NK cells (G and H); and CD69+ NK cells (I and J) per spleen were measured from WT B6 and TLR5 KO B6 mice. K–P. Numbers of: CD11b+ NK cells (K); CD122+ NK cells (L); 2B4+ NK cells (M); Ly49G2+ NK cells (N); Ly49C/H+ NK cells (O); and Ly49D+ NK cells (P) per spleen were determined from rflagellin- and PBS-treated WT B6 mice on day 0 and 3 after mCMV infection. The “*” and “**” represent p values<0.05 and <0.005, respectively, Students *t*-test. Q. mRNAs were harvested from the TLR5-transfected and TLR5-ve Null cells and FACS-sorted splenic CD3-NK1.1+ NK cells from naïve WT B6 mice as described in Materials and Methods. The cDNA bands specific for TLR5 and GAPDH were measured by RT-PCR and were visualized by ethidium bromide staining. Lane 1 = TLR5 expressing cells, Lane 2 = FACS sorted NK cells, and Lane 3 = TLR5-ve Null cells.

The peak number of activated cytotoxic NK cells is typically seen in the spleen 2–3 days after infection [26]. To explore the role of NK cells against mCMV infection in rflagellin-treated mice, we next analyzed the anti-mCMV immunity of NK cells in the spleen of rflagellin-treated WT mice on day 0 and 3 after mCMV infection (2 and 5 days after rflagellin administration). To confirm the role of TLR5-signaling in anti-mCMV immunity of NK cells after rflagellin administration, we studied anti-mCMV immunity of NK cells in rflagellin-treated and mCMV-infected TLR5 KO mice. Total numbers of splenocytes were significantly increased 2 days after rflagellin-treatment (day 0 after mCMV infection) in WT mice compared with PBS-treated control mice, with the greatest effect seen 3 days following mCMV infection (Figure 4A). Surprisingly, rflagellin-treated TLR5 KO mice had significantly increased numbers of splenocytes 2 days (day 0 mCMV infection) after rflagellin-treatment compared to PBS-treated TLR5 KO mice, but no differences in splenocyte numbers were detected 3 days after mCMV infection (Figure 4B). The numbers of splenic CD3-NK1.1+ NK cells, KLRG1+, ICOS-1+ and CD69+ activated NK cells were significantly higher on both days 0 and 3 after mCMV infection in rflagellin-treated WT B6 mice compared with PBS-treated control mice (Figure 4C, 4E, 4G and 4I). While rflagellin treatment resulted in an increase in the numbers of total splenic NK cells and KLRG1+ NK cells 48 hours later in TLR5 KO mice, there was no significant effect on numbers of NK cell subsets or ICOS-1+ or CD69+ NK cells by day 3 after MCMV infection compared with the PBS-treated TLR5 KO control mice (Figures 4D, 4F, 4H and 4J). However, administration of 25 µg highly purified native flagellin 2 days prior to mCMV infection in TLR5 KO mice did not have any effect on NK cells in spleen in contrast to significantly increased numbers of splenic NK cells and KLRG1+ NK cells in WT mice (Figure S4). We next determined the expression of other activation and/or inhibitory markers on NK cells harvested from the spleen of rflagellin-treated and mCMV-infected WT B6 mice [22], [30]–[32]. The numbers of CD11b+, CD122+, 2B4+, Ly49G2+, Ly49C/H+ and Ly49D+ NK cells were significantly increased in the spleen 2 days after rflagellin treatment with these differences persisting (with lower absolute numbers of NK cells) 3 days later following mCMV infection compared with the PBS-treated control mice (Figure 4K to 4P). To determine whether rflagellin activated NK cells in mice through direct or indirect pathways, we next examined the TLR5 expression on NK cells by RT-PCR. FACS-sorted CD3-NK1.1+ NK cells harvested from naïve B6 mice did not express TLR5 (Figure 4Q lane 3), validated by using the TLR5 transfected cell lines (Figure 4S lane 1) and TLR5 negative cell lines (Figure 4S lane 2). These data suggest that rflagellin-TLR5 interactions indirectly activate NK cells.

**Figure 5:**
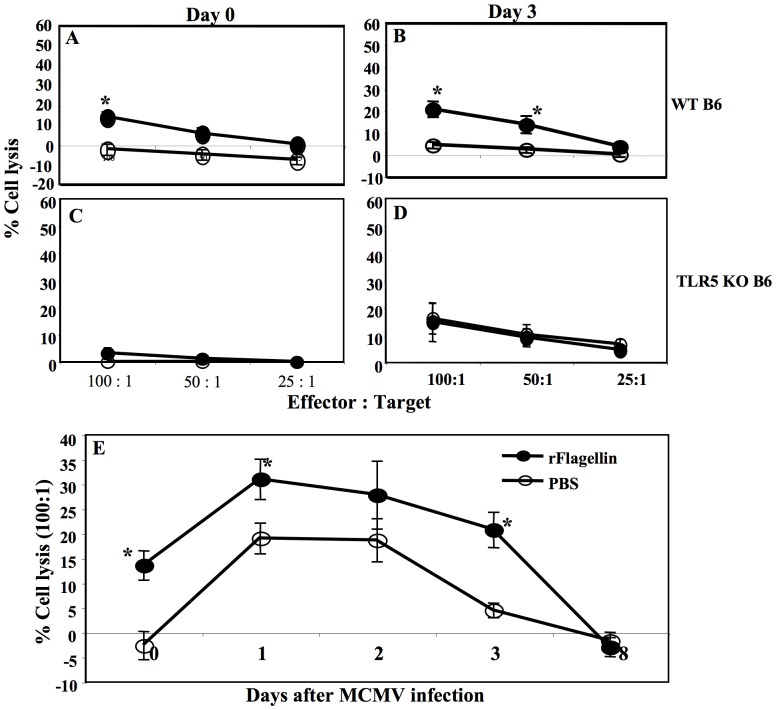
rFlagellin increased NK cell lytic activity in the presence and absence of mCMV infection. Splenocytes were harvested on days 0 and 3 after mCMV infection and NK cell lytic activity was measured using standard 4-hour ^51^Cr-release assay by Yac-1 target cells as described in Materials and Methods. A and B. The % cell lytic activity of NK cells of rflagellin- and PBS-treated WT B6 mice on days 0 and 3 after infection. C and D. The % cell lytic activity by NK cells harvested from rflagellin- and PBS-treated TLR5 KO B6 mice on day 0 and 3 post infection. E. Kinetics of NK cells lytic activity from the splenocytes harvested from rflagellin and PBS-treated WT B6 mice on day 0, 1, 2, 3 and 8 after mCMV infection. The data shown in A–D are representative of three independent experiments and data shown in E are from one experiment. 5 mice were used per group per time point. The “*” indicates p value<0.05, Student's T-test.

Quantitative anti-mCMV activity of NK cells against mCMV infection is generally determined by measuring the cytolytic activity of NK cells *ex vivo* using ^51^Cr-pulsed Yac-1 target cells [26], [33], [34]. We therefore determined the cytolytic activity of NK cells against ^51^Cr-pulsed Yac-1 target cells in splenocytes harvested from the rflagellin- and PBS-treated WT or TLR5 KO mice on days 0 and 3 after mCMV infection. As expected, NK-cell cytolytic activity was significantly increased in the spleen of rflagellin-treated WT mice 48 hours after rflagellin treatment and also on day 3 after mCMV infection compared with the PBS-treated control mice (Figure 5A and 5B). There was no difference in NK cell lytic activity in rflagellin-treated TLR5 KO mice compared with PBS-treated TLR5 KO mice (Figure 5C and 5D).

To explore the time to peak NK lytic activity in rflagellin-treated WT mice, we next determined the kinetics of NK cell lytic activity in the spleens of both rflagellin- and PBS-treated WT mice on days 0, 1, 2, 3 and 8 after mCMV infection. As expected, significantly higher levels of NK lytic activity were detected 48 hours after rflagellin-treatment, and 1 and 3 days after mCMV infection in rflagellin-treated mice compared with the PBS-treated mice, while no cytolytic activity was detected on day 8 after mCMV infection in either group of mice (Figure 5E). Collectively, these data suggest that rflagellin transiently enhances the activation and cytolytic activity of NK cells, and that pre-treatment with rflagellin 2 days prior to mCMV infection results in optimal anti-mCMV activity of NK cells.

**Figure 6:**
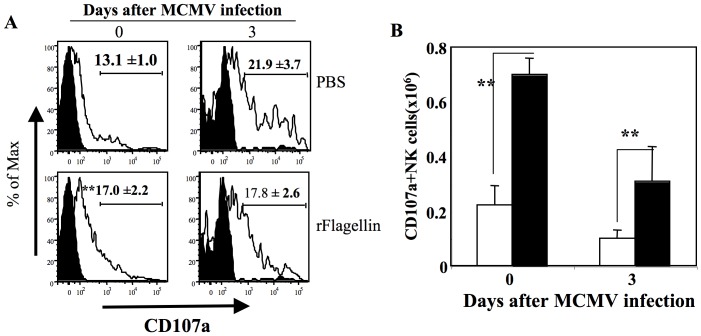
rFlagellin enhanced NK cells cytotoxicity by increasing CD107a degranulation in NK cell. Splenocytes were harvested from rflagellin- and PBS-treated mice on days 0 (48 hours after rflagellin treatment) and 3 after mCMV infection (1×10^5^ pfu/mouse i.p). Flowcytometric analysis for CD107a expressed by NK cells was performed by both regular surface stainings as described in Materials and Method. A. The representative FACS data of CD3-NK1.1+ NK cells expressing CD107a in the spleen of rflagellin- and PBS-treated mice determined by regular surface staining. B. The absolute numbers of CD107a+NK cells per spleen were determined from rflagellin- and PBS-treated mice on day 0 (48 hours after rflagellin treatment) and 3 after mCMV infection by using the flowcytometric data. 5 mice were used per group per time point. The “*” and “**” represent the p values<0.05 and <0.005, respectively, Student's T-test.

The increased killing of NK sensitive Yac-1 target cells by splenocytes harvested from the rflagellin-treated WT mice could be due to either increased numbers of activated NK cells or increased cytolytic activity per cell. In absence of mCMV infection rflagellin-treated splenocytes harvested from the WT mice had <2-folds increase numbers of NK cells per spleen compare with the PBS-treated splenocytes (Figure 4C). But the rflagellin-treated splenocytes showed Yac-1 target cells killing activity (13.6%±3.1% at 100∶1 effector/target ratio) whereas PBS-treated splenocytes showed (−2.6%±2.1% at 100∶1 effector/target ratio) which is at least >13 times more (even considering PBS-treated splenocytes killing effect 0–1% at 100∶1 effector/target ratio) (Figure 5A). These data indicated that rflagellin treatment enhanced the lytic activity of NK cells in addition to increasing the total numbers of NK cells in the spleen. A number of previously published studies have shown that cytotoxic activity of NK cells is directly related to the degranulation of lysosomal-associated membrane protein-1 (LAMP-1 or CD107a) by NK cells [18], [35], [36]. To further confirm the increased lytic activity of NK cells in rflagellin-treated mice, we next measured the degranulation of CD107a in NK cells harvested from the spleens of rflagellin- and PBS-treated mice 2 days after rflagellin treatment (day 0 after mCMV infection) and 3 days after mCMV infection. NK cells from rflagellin-treated mice expressed increased levels of surface CD107a 2 days following rflagellin treatment (Figure 6A). In contrast, by day 3 after mCMV infection a larger proportion of NK cells had degranulated, and differences were not seen in comparing NK cells from PBS- to rflagellin-treated mice (Figure 6A). However, significantly higher numbers of CD107a+ NK cells per spleen were determined on both day 0 and 3 after mCMV infection in rflagellin-treated mice compared with the PBS-treated mice (Figure 6B). These data suggest the direct evidence of increased cytolytic activity of NK cells following rflagellin treatment.

### rFlagellin enhanced NK cytolytic activity through increasing the numbers of mature Ly49H+ NK cells

**Figure 7:**
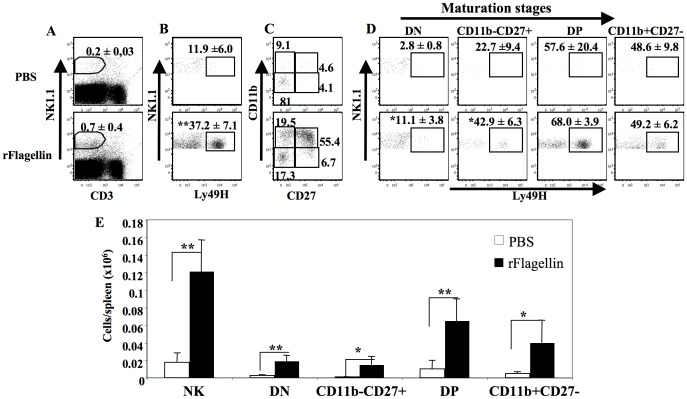
rFlagellin treatment enhanced maturation and increased expression of Ly49H on NK cells after mCMV infection. Harvested splenocytes on day 3 after mCMV infection from rflagellin- and PBS-treated control mice were stained with mAbs to NK1.1, CD27, CD11b along with Ly49H as described in Materials and Methods. A. FACS plots of % CD3-NK1.1+ NK cells of lymphocyte-gated populations. B. % Ly49H expressed by NK cells. C. CD11b−CD27−(DN), CD11b−CD27+, CD11b+CD27+ (DP) and CD11b+CD27− NK cell populations. D. Ly49H+ NK cells of 4 subsets gated populations described in C. E. The total numbers of Ly49H+ NK cells and all 4 subsets of NK cells (as described in D) per spleen expressed Ly49H on day 3 after mCMV infection. The “*” represents p value<0.05, Student's T-test. 5 mice were used per group.

A 4-stage model of NK cell maturation pathways associated with increased effector function has been described based on the expression of CD11b and CD27. The suggested sequence of maturation stages of NK cells is: CD11b^−^CD27^−^ (double negative, DN)→CD11b^−^CD27^+^→CD11b^+^CD27^+^ (double positive, DP)→CD11b^+^CD27^−^ [30]. Additionally, mCMV infection increases the expression of Ly49H on activated cytotoxic effector NK cells and specifically enhances killing of mCMV-infected target cells *in vivo* [35]. We next investigated the effect of rflagellin treatment on the numbers of Ly49H-expressing NK cells and the frequencies of the 4 maturation stages of Ly49H-expressing NK cell subpopulations on day 3 after mCMV infection. Although the percentage of CD3-NK1.1+ NK cells was higher (but statistically insignificant) in rflagellin-treated mice compared with PBS-treated mice (Figure 7A), the percentages of Ly49H+ NK cells increased significantly on day 3 after MCMV infection in rflagellin-treated mice compared with the PBS-treated control mice (Figure 7B). rFlagellin treatment increased the frequencies of CD11b^−^CD27^+^, DP and CD11b^+^CD27^−^ NK cell subpopulations on day 3 after mCMV infection compared with the PBS-treated mice (Figure 7C). The percentages of cells expressing Ly49H increased significantly in the DN and CD11b-CD27+ subsets of NK cells, but not the more mature DP and CD11b+ CD27− subsets, in rflagellin-treated mice on day 3 after mCMV infection compared with the PBS-treated mice (Figure 7D). However, the absolute numbers of Ly49H+NK cells and all 4 maturation subsets of Ly49H+NK cells per spleen were significantly increased in rflagellin-treated mice compared with PBS-treated mice on day 3 following mCMV infection (Figure 7E). These data suggest that rflagellin treatment enhanced NK cell maturation, and upregulated Ly49H expression on all NK cell subsets following mCMV infection.

### rFlagellin enhanced IFN-γ and granzyme B producing NK cells

**Figure 8:**
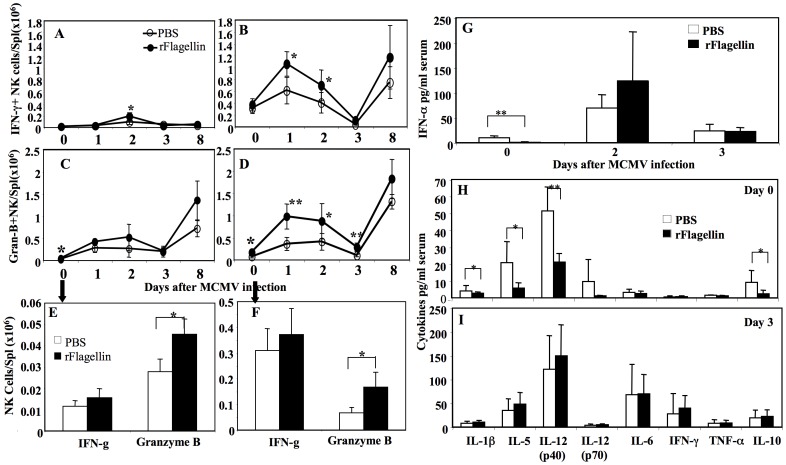
The effect of rflagellin treatment on cytokine production in the presence and absence of mCMV infection. Splenocytes harvested from rflagellin- and PBS-treated control mice on day 0, 1, 2, 3 and 8 after mCMV infection were stimulated with PMA ionomycin for 4 hours at 37°C as described in Materials and Methods. Cells were stained for intracellular expression of IFN-**γ** and granzyme B along with NK cell surface markers. A and B represent the numbers of IFN-**γ** producing NK cells per spleen in the absence and presence of PMA-ionomycin stimulation. C and D represent the numbers of granzyme B producing NK cells per spleen in the absence and presence of PMA ionomycin stimulation. E and F represent the numbers of IFN-**γ** and granzyme B producing NK cells per spleen in the absence and presence of PMA ionomycin stimulation, harvested on day 0 after mCMV infection. G. Serum IFN-α on day 0, 2 and 3 after mCMV infection determined by Luminex assay. H and I represent serum cytokines determined on day 0 and 3 after mCMV infection, determined by Luminex assay. 5 mice were used per group in each time point. The “*” and “**” represent p values<0.05 and <0.005, respectively, Student's T-test.

We previously showed that highly purified native flagellin reduced GvHD in allo-BMT recipients through reduced production of IFN-γ, TNF-α and IL-6 during the first 10 days post-transplant [8]. Since the anti-MCMV immunity of NK cells is mostly controlled by a set of cytokines/chemokines induced by mCMV infection [33], [34], [37], [38], we first measured the numbers of IFN-γ and granzyme B-producing splenic NK cells in rflagellin-treated mice following *in vitro* culture in media with brefeldin A alone or following stimulation with PMA-ionomycin plus brefeldin A. Compared with the PBS-treated control mice, the numbers of IFN-γ producing NK cells in rflagellin-treated mice were significantly higher on day 2 after mCMV infection without stimulation (Figure 8A) and on both days 1 and 2 after mCMV infection following PMA-ionomycin stimulation (Figure 8B). The numbers of splenic granzyme B+ NK cells were significantly higher in rflagellin-treated mice on day 0 post mCMV infection without stimulation, (Figure 8C and 8E) and on day 1, 2 and 3 post mCMV infection after PMA-ionomycin stimulation compared with the PBS-treated control mice (Figure 8D and 8F). These data suggest that increased anti-mCMV activity of NK cells in rflagellin-treated mice is mediated by increased number of IFN-γ and granzyme B-producing NK cells.

### rFlagellin reduced production of pro-inflammatory cytokines in the absence of CMV infection

Besides NK cells, antigen-presenting cells (APCs), epithelial cells, and endothelial cells produce cytokines/chemokines which directly or indirectly control anti-mCMV immunity of NK cells [37]. We next compared the levels of serum cytokines and chemokines in rflagellin-treated versus PBS-treated mice. Serum harvested from the rflagellin-treated mice had significantly reduced levels of IFN-α (but not IFN-β) 48 hours after treatment compared with the PBS-treated control mice, but similar levels of IFN-α (and IFN- on days 2 and 3 after MCMV infection (Figure 8G)). Moreover, we measured significantly reduced serum levels of IL-1, IL-5, IL-12p40 (not IL-12p70) and IL-10 on 48 hours after rflagellin treatment compared with the PBS-treated control mice (Figure 8H). In contrast, there were no differences in levels of any of the 26 cytokines/chemokines tested on day 3 after mCMV infection between rflagellin- and PBS-treated mice (only representative pro-inflammatory cytokines are shown in Figure 8I). Collectively, these data suggest that rflagellin administration reduces production of pro-inflammatory cytokines and IFN-α without decreasing the activation and maturation of NK cells (Figure 4 and 5A).

## Discussion

Using an established mouse model of mCMV infection, we have explored the mechanism by which rflagellin enhances anti-mCMV immunity of NK cells. This study is an important extension of our previously reported work focused on the pre-clinical use of rflagellin to reduce GvHD and opportunistic infections. We have shown that administration of highly purified native flagellin reduced GvHD in murine allo-BMT recipients and protects against mCMV infection [8] and that peritransplant administration of either 25 µg or 50 µg rflagellin resulted in a comparable reduction in the severity of GvHD (unpublished data). In the current studies, we therefore used a single intraperitoneal dose of 25 µg rflagellin to elucidate the mechanism by which rflagellin enhances anti-mCMV immunity. We observed that prophylactic administration of rflagellin protected WT B6 mice from lethal mCMV infection, and that optimization of anti-mCMV immunity depends on the timing of rflagellin administration. Administration of rflagellin 48 hours before mCMV infection led to significantly decreased viral load that was associated with increased numbers of mature, activated cytotoxic NK cells without a concomitant increase in pro-inflammatory cytokines.

These data indicate that tonic signaling by rflagellin through TLR5 is required for optimal activation of NK cells in response to mCMV infection. The activation of NK cells by rflagellin in TLR5 KO mice was functionally incomplete as increased cytolytic activity was not seen compared with NK cells from PBS-treated TLR5 KO mice (Figure 4C & 4D) and the NK cells in TLR5 KO mice did not upregulate the activation markers CD69 and ICOS-1 following rflagellin treatment and MCMV infection (Figure 4H and 4J). While mCMV-infected TLR5 KO mice had >10-fold higher titers of virus in the liver compared with WT B6 mice on day 3 after mCMV infection, TLR5 KO mice successfully recovered from mCMV infection (Figure 2), indicating that other non-TLR5-dependent pathways activate and initiate anti-mCMV innate immunity [6].

This study also indicates a role for non-TLR5 dependent signaling in response to rflagellin. We observed increased numbers of NK cells and KLRG1+ NK cells in the spleen of rflagellin-treated TLR5 KO mice 48 hours after treatment (Figure 4B, 4D and 4F). These data suggest that rflagellin may activate NK cells through a TLR5-independent pathway, consistent with prior reports of both TLR5-dependent and independent pathways of flagellin-induced immune responses [39], [40]. In addition to direct flagellin-TLR5 interactions, intracellular flagellin also binds with the cytosolic immunosurveillance proteins NLR (nucleotide-binding domain, leucine-rich repeat)-containing apoptosis inhibitory proteins, NAIPs, in a TLR5-independent pathway [39], [40].

Ly49H is a CMV-1 encoded NK cell-activating receptor that specifically recognizes the m157 viral protein on the surface of mCMV-infected cells in association with DAP12 adaptor protein complex [19]–[23], and *in vivo* depletion of Ly49H by mAb in mCMV infection has been reported to increase viral titers in infected organs [35]. 2B4, also known as CD244, is a non-MHC binding receptor that also activates NK cells against mCMV infection by coupling with NKG2D-DAP10 adaptor complex molecules [19].

Our data indicate that up-regulation of the Ly49H and 2B4 surface proteins are the predominant mechanism underlying rflagellin-enhanced NK cell immunity against mCMV infection (Figure 4M and 4O) [19], [41]. Additionally, rflagellin treatment enhanced the expression of activation markers on NK cells and also increased the numbers of NK cells in the spleens of WT B6 mice (Figure 4). Cytotoxic activity of NK cells is directly related to the degranulation of lysosomal-associated membrane protein-1 (LAMP-1 or CD107a) by NK cells [18], [35], [36] and we also measured significantly increased numbers of CD107a degranulation in NK cells in rflagellin- treated mice compared with PBS-treated mice both in absence and presence of mCMV infection (Figure 6). Therefore, the increased killing of NK sensitive Yac-1 target cells by splenocytes from rflagellin-treated mice is due to increased numbers of activated NK cells as well as increased cytotoxic activity of individual NK cells.

A relevant question related to this work is whether the administration of rflagellin could have clinical utility in patients at risk for opportunistic viral infections such as allo-BMT recipients. We have shown that a single dose of rflagellin administered 48 hours before mCMV infection enhanced anti-mCMV immunity, and administration of rflagellin at other times relative to mCMV infection had a reduced positive effect on anti-mCMV immunity (Figure 1A and 1D). Thus, the optimal schedule of rflagellin administration, 2 days before viral infection, might preclude its clinical application as treatment or prophylaxis for mCMV infection. However, administration of rflagellin significantly reduced production of inflammatory cytokines on day 0 of mCMV infection (Figure 8G and H), and peritransplant administration of native flagellin reduced GvHD and also reduced inflammatory cytokines in allo-BMT recipients [8]. Since inflammatory cytokines have been directly correlated with GvHD pathogenesis [42]–[44], early post-transplant administration of rflagellin in allo-BMT could provide clinical benefit in allo-BMT recipients by enhancing NK cell activity [45] without increasing the risk of GvHD. Alternatively, ex vivo treatment of NK cells with rflagellin might be useful to generate activated NK cells that could be used as adoptive cellular therapy in patients, as human NK cells express TLR5 [27]. Recently published data showed that repeated subcutaneous administration of very low dose rflagellin (e.g, 2 µg/mouse) in allo-BMT recipients enhanced anti-tumor immunity of CD8+ T cells without increasing GvHD toxicity [46]. The use of repeated administration of much lower doses of rflagellin during mCMV infection in non-transplant settings as well as in the peritransplant period in allo-BMT recipients may enhance anti-mCMV immunity, and these experimental approaches are currently under investigation in our lab.

In summary, we have shown that administration of a single dose of rflagellin significantly enhanced innate immunity by increasing the activation status and cytotoxic activity of NK cells against mCMV-infected targets. The rflagellin used in this study is a pharmacologically optimized TLR5 agonist that is less toxic and less immunogenic than native flagellin, and is currently being evaluated in clinical trials as a vaccine adjuvant. The results from this study provide mechanistic insights that may be exploited for clinical benefit using rflagellin to reduce opportunistic infections in immune-compromised patients.

## Supporting Information

Figure S1 rFlagellin did not induce noticeable toxicity in mice. WT B6 mice were treated with 25 µg rflagellin/mouse i.p 48 hours before a sub-lethal dose (1×10^5^ pfu/mouse i.p) of mCMV infection. Weight and overall physical activity of individual mouse were recorded as parameters of rflagellin toxicity. A). Weight loss of rflagellin-treated mice (n = 20) was determined by measuring weight on 0 and 48 hours after rflagellin treatment. No signs of physical sickness in rflagellin-treated mice after 48 hours of injection. B). Weight loss of rflagellin-treated individual mouse (n-9) was determined by measuring weight on 0 and 3 days after MCMV infection. C). Weight loss of rflagellin-treated mice (n-8) was determined by measuring weight of individual mouse on 0 and 3 days after MCMV infection. The “*” represents p values<0.05, Student's T-test.
(TIFF)

Figure S2 TLR5 KO B6 mice are more susceptible to mCMV infection than WT B6 mice. Four groups of WT B6 and TLR5 KO B6 mice were infected with 2.5×10^5^ pfu/mouse, 5×10^5^ pfu/mouse, 1×10^6^ pfu/mouse or 2.5×10^6^ pfu/mouse i.p mCMV. Survival of infected mice was monitored by recording and weight every day. Mice having >25% weight loss were euthanized and included in the list of mortality. A. Percent survival of WT B6 mice data. B. Percent survival of TLR5 KO B6 mice data. 5–10 mice were used per group. C. The LD50 of WT B6 mice and TLR5 KO B6 mice against mCMV infection were calculated from the survival data of Figure A and B.
(TIFF)

Figure S3 Treatment of anti-asialo GM1 caused >99% *in vivo* NK cell depletion. 0.5 ml of reconstituted anti-asialo GM1 in PBS were injected to B6 mice on −4, −3 and −1 days of mCMV infection as described in Materials and Methods and in Figure 3. Control WT B6 mice were injected with 0.5 ml PBS. 25 µg rflagellin/mouse i.p was injected 48 hours before mCMV infection in anti-asialo GM1-treated and or PBS treated WT B6 mice. Representative two mice from PBS-treated control group, two mice from anti-asialo GM1-treated group and one mouse from anti-asialo GM1 and rflagellin-treated group were bled before mCMV infection. Depletion of NK cells in blood was determined by flowcytometry.
(TIFF)

Figure S4 Administration of native flagellin had no effect on NK cells in TLR5 KO mice. WT B6 and TLR5 KO B6 mice were treated with highly purified native flagellin (25 µg/mouse i.p) extracted from the *S. typhimurium*. Control mice were injected with 0.2 ml PBS i.p. 48 hours later both native flagellin- and PBS-treated mice were sacrificed and splenocytes were harvested. The numbers of nucleated cells per spleen were determined by counting the cells under microscope. The numbers of NK cell and KLRG1+ NK cells were determined by FACS. 5 mice were used per group. The “**” represents p values<0.005, Student's T-test.
(TIFF)
