# Supplementary figures and images for: Recombinant TLR5 Agonist CBLB502 Promotes NK Cell-Mediated Anti-CMV Immunity in Mice
Source: PLoS One. 2014 May 30;9(5):e96165. doi: 10.1371/journal.pone.0096165 (PMC4039429; doi:10.1371/journal.pone.0096165)

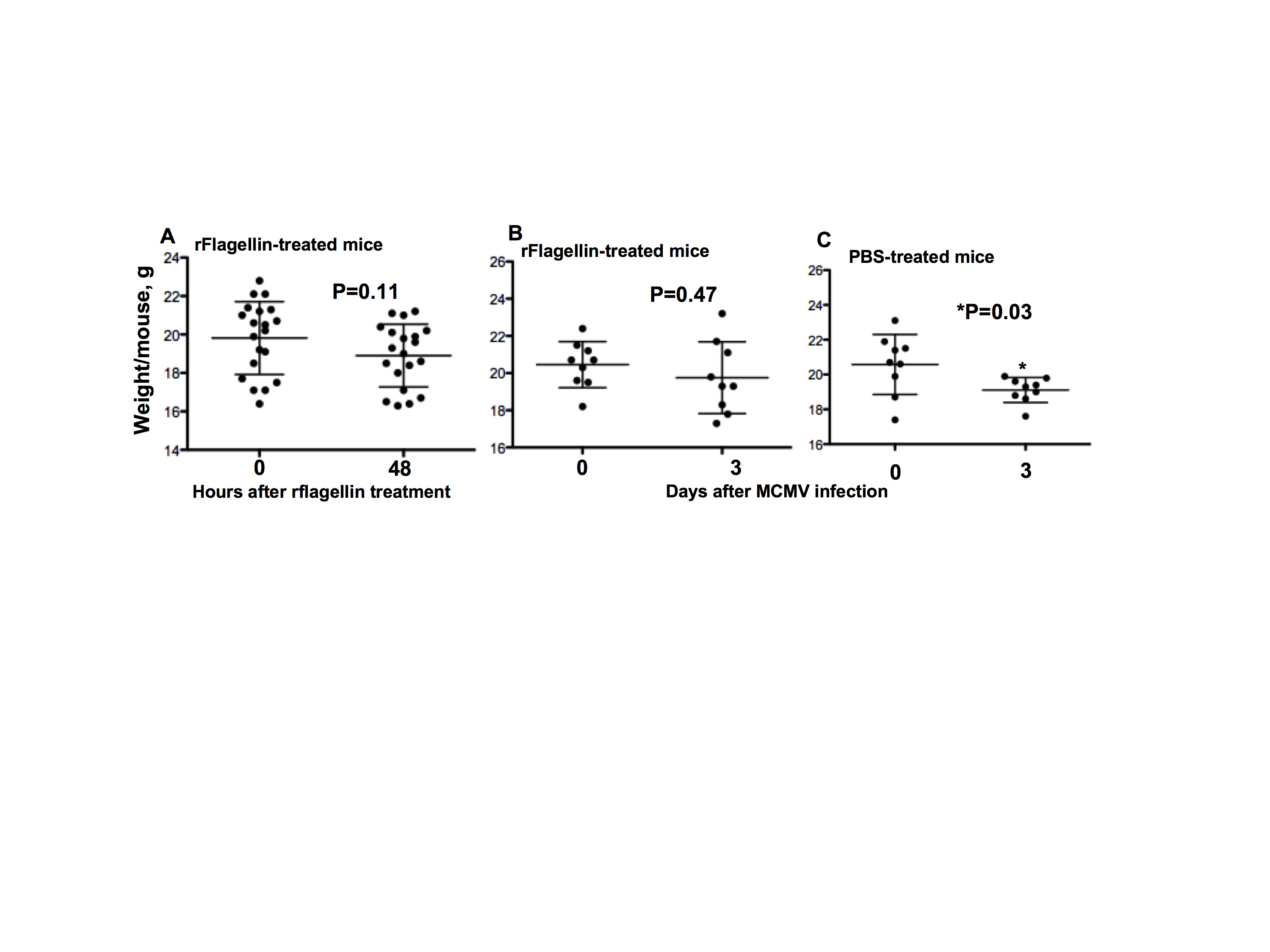

Supplement: Figure S1 — rFlagellin did not induce noticeable toxicity in mice. WT B6 mice were treated with 25 µg rflagellin/mouse i.p 48 hours before a sub-lethal dose (1×10^5^ pfu/mouse i.p) of mCMV infection. Weight and overall physical activity of individual mouse were recorded as parameters of rflagellin toxicity. A). Weight loss of rflagellin-treated mice (n = 20) was determined by measuring weight on 0 and 48 hours after rflagellin treatment. No signs of physical sickness in rflagellin-treated mice after 48 hours of injection. B). Weight loss of rflagellin-treated individual mouse (n-9) was determined by measuring weight on 0 and 3 days after MCMV infection. C). Weight loss of rflagellin-treated mice (n-8) was determined by measuring weight of individual mouse on 0 and 3 days after MCMV infection. The “*” represents p values<0.05, Student's T-test. (TIFF) [file pone.0096165.s001.tiff]

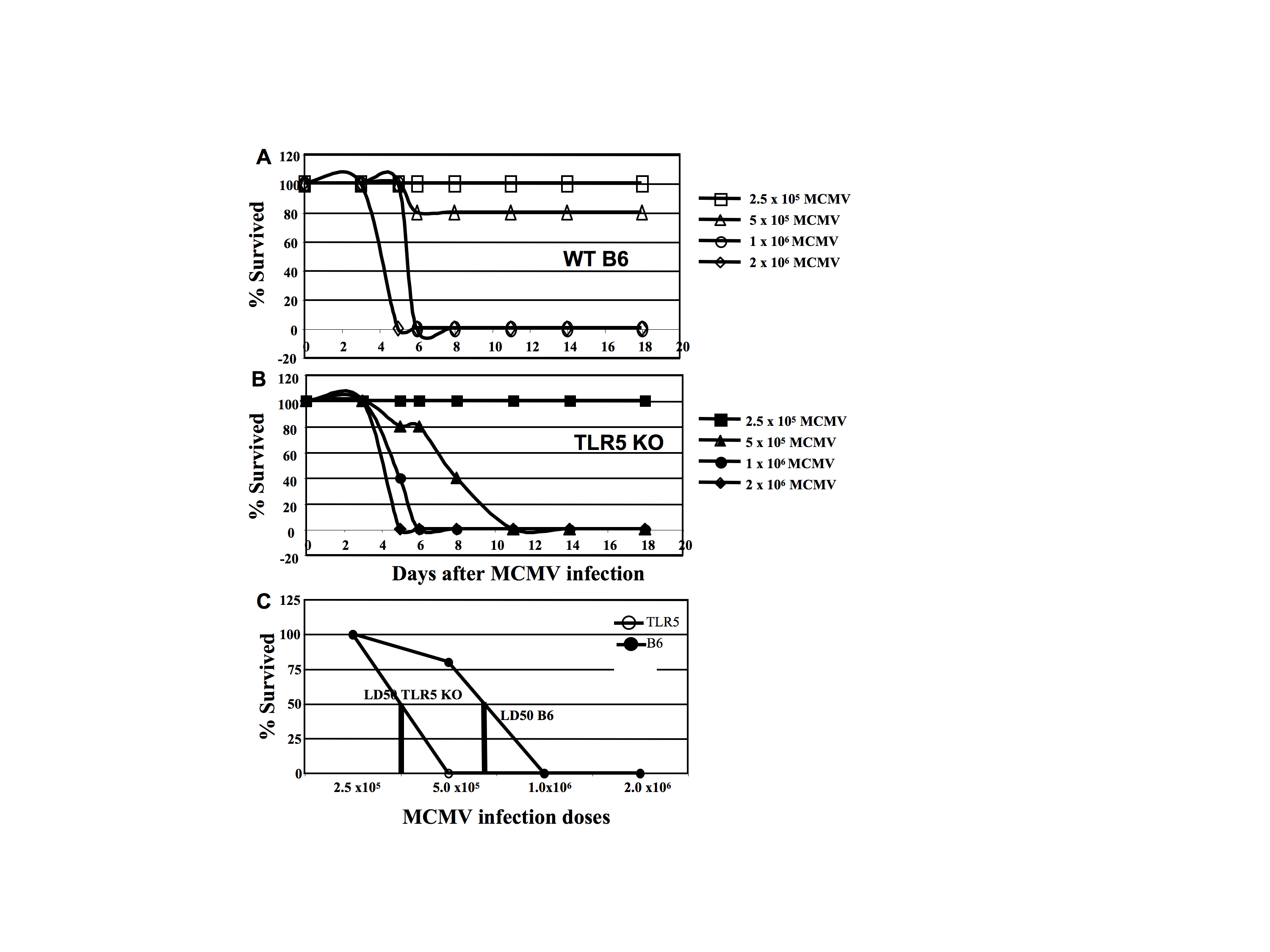

Supplement: Figure S2 — TLR5 KO B6 mice are more susceptible to mCMV infection than WT B6 mice. Four groups of WT B6 and TLR5 KO B6 mice were infected with 2.5×10^5^ pfu/mouse, 5×10^5^ pfu/mouse, 1×10^6^ pfu/mouse or 2.5×10^6^ pfu/mouse i.p mCMV. Survival of infected mice was monitored by recording and weight every day. Mice having >25% weight loss were euthanized and included in the list of mortality. A. Percent survival of WT B6 mice data. B. Percent survival of TLR5 KO B6 mice data. 5–10 mice were used per group. C. The LD50 of WT B6 mice and TLR5 KO B6 mice against mCMV infection were calculated from the survival data of Figure A and B. (TIFF) [file pone.0096165.s002.tiff]

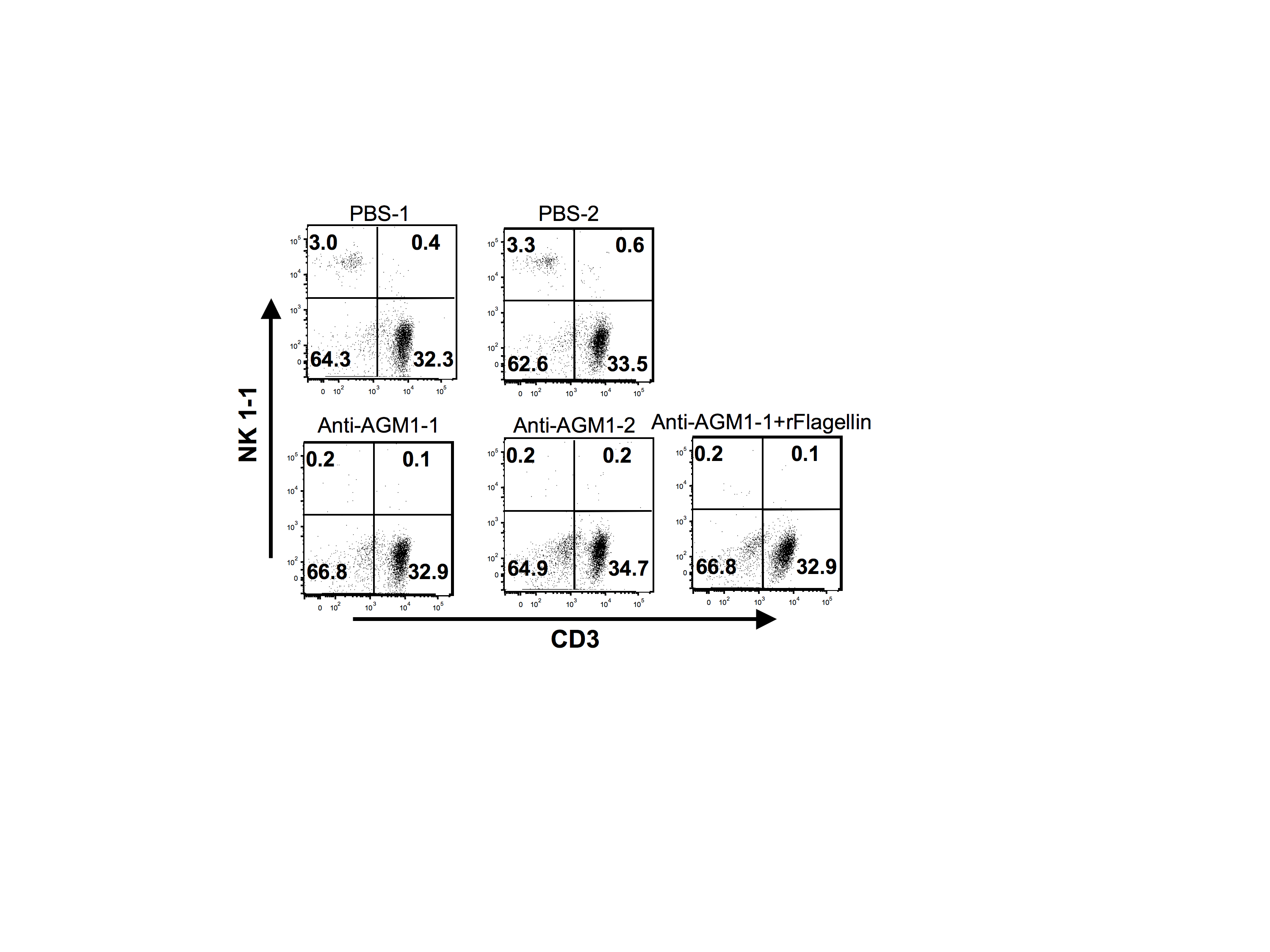

Supplement: Figure S3 — Treatment of anti-asialo GM1 caused >99% in vivo NK cell depletion. 0.5 ml of reconstituted anti-asialo GM1 in PBS were injected to B6 mice on −4, −3 and −1 days of mCMV infection as described in Materials and Methods and in Figure 3. Control WT B6 mice were injected with 0.5 ml PBS. 25 µg rflagellin/mouse i.p was injected 48 hours before mCMV infection in anti-asialo GM1-treated and or PBS treated WT B6 mice. Representative two mice from PBS-treated control group, two mice from anti-asialo GM1-treated group and one mouse from anti-asialo GM1 and rflagellin-treated group were bled before mCMV infection. Depletion of NK cells in blood was determined by flowcytometry. (TIFF) [file pone.0096165.s003.tiff]

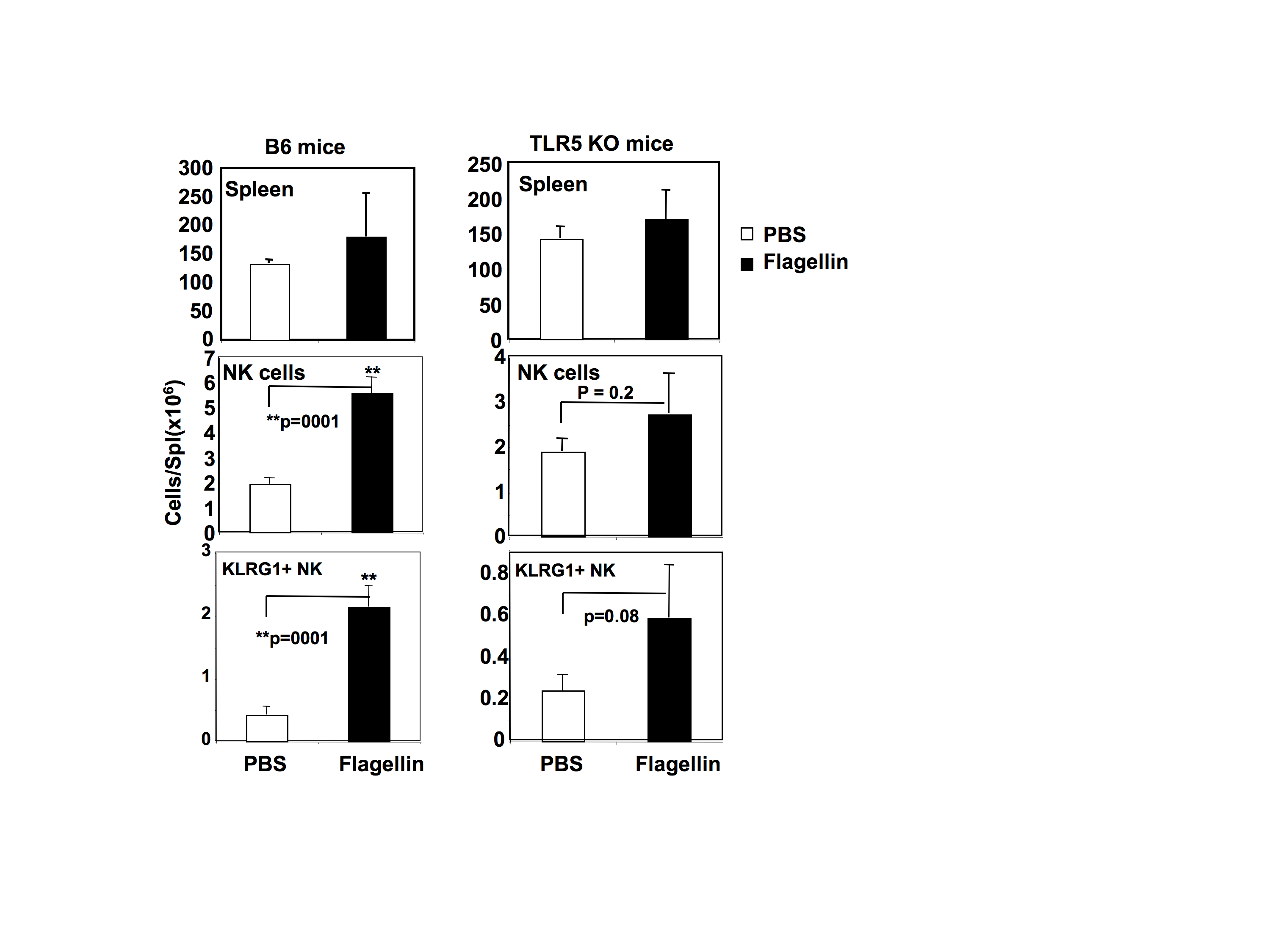

Supplement: Figure S4 — Administration of native flagellin had no effect on NK cells in TLR5 KO mice. WT B6 and TLR5 KO B6 mice were treated with highly purified native flagellin (25 µg/mouse i.p) extracted from the S. typhimurium. Control mice were injected with 0.2 ml PBS i.p. 48 hours later both native flagellin- and PBS-treated mice were sacrificed and splenocytes were harvested. The numbers of nucleated cells per spleen were determined by counting the cells under microscope. The numbers of NK cell and KLRG1+ NK cells were determined by FACS. 5 mice were used per group. The “**” represents p values<0.005, Student's T-test. (TIFF) [file pone.0096165.s004.tiff]
